# Rescued by His Horse

**Published:** 1892-05

**Authors:** 


### RESCUED BY HIS HORSE.

About five miles south of Plattsmouth, Neb., is one of the most dangerous pieces of road in the country, and while in some seasons of the year it is safe for travelling, at others it is all a man's life is worth to go over it. The cause of this is a quicksand which lies directly across the road, which is only used in the fall of the year and abandoned during the spring and summer. Everybody in this part of the country is acquainted with the roads and their peculiarities, but to prevent strangers from getting into trouble a signboard is generally placed at the forks to warn them about the quicksand. In some manner that sign was removed, and the result was that Henry Girard, who lives near Joplin, Mo., came near losing his life. He is now in the hotel in this city, where he is slowly recovering from his terrible experience. Girard tells the following story:

I was coming to Plattsmouth to transact some business regarding some lands in which I have an interest, and as I wished to see the land before I got there, I concluded to come on horseback. I had plenty of time and rode all the way from home on my horse, to which fact I to-day owe my life. The horse is one that I raised myself, and he is a regular pet, coming at my call. When I got to the place where the roads forked I saw that the river road leading up the steep hill was the one which seemed to be traveled. I did not like to go over the hill unless I had to, so I got down off my horse and turned him loose to follow me as I walked along. He stopped to graze and I walked slowly, watching the river. Having got some distance ahead of the horse, I sat down to wait for him.

How long I sat there I do not know, but I was suddenly aroused by finding that my legs were firmly grasped by the quicksand and that I was slowly but surely being drawn into the earth. I threw myself down on my back and tried to pull myself from the sand, but all my efforts were unavailing, and I continued to sink. I shouted for help until I was almost exhausted, but could get no response, and was forced to believe that my last hour had come and that I was to suffer a most horrible form of death. It was late in the evening, and as the darkness came I wondered why my horse did not come, and called him time and again; but he evidently was too far away to hear me.

As I lay there sinking deeper and deeper every minute I heard a party of people on the river. It appeared to be a pleasure party of young folks, and as they floated down the stream they were singing. Their song came distinctly floating over the water, but for some reason I could not make them hear, and they passed on down, singing "Home, Sweet Home," while I lay there thinking that I had seen my home for the last time.

By this time the sand had pulled me down until I was almost up to my shoulders and I lay with my arms spread out, in order to get as much resistance to the terrible suction as possible. I thought I heard my horse, and called with all my might, giving a peculiar whistle which I had taught him to answer. He heard me and came running to where I lay, but could not see me on the ground, as it was by this time very dark. I called him again and again until he at last found where I was, and then I tried to reach up, and get some sort of hold by which he could pull me out, but I was too low. By some chance he stepped past me and as he did so I got a firm hold on his tail and then urged him on. It was an awful pull, but I could feel that I was being dragged out of the sand. I strained my arms so that I was compelled to stop and rest often, but at last I was drawn out so that I could extricate myself, and then managed to crawl away from the spot where I had undergone such terrible mental and physical suffering. I was afraid to stay any where near the place, and crawled back to the forks of the road, where I lay with the horse near me until a wagon came along and I was brought here.—*Ex.*

